# Functional priorities reported by parents of children with cerebral palsy: contribution to the pediatric rehabilitation process

**DOI:** 10.1590/bjpt-rbf.2014.0064

**Published:** 2014

**Authors:** Marina B. Brandão, Rachel H. S. Oliveira, Marisa C. Mancini

**Affiliations:** 1 Núcleo de Ensino e Pesquisa, Associação Mineira de Reabilitação (AMR), Faculdade de Ciências Médicas de Minas Gerais, Belo Horizonte, MG, Brazil; 2 Curso de Terapia Ocupacional, Núcleo de Ensino e Pesquisa, AMR, Faculdade de Ciências Médicas de Minas Gerais, Belo Horizonte, MG, Brazil; 3 Programa de Pós-graduação em Ciências da Reabilitação, Departamento de Terapia Ocupacional, Universidade Federal de Minas Gerais (UFMG), Belo Horizonte, MG, Brazil

**Keywords:** functional priorities, cerebral palsy, rehabilitation, children

## Abstract

**Background:**

Collaborative actions between family and therapist are essential to the rehabilitation process, and they can be a catalyst mechanism to the positive outcomes in children with cerebral palsy (CP).

**Objectives:**

To describe functional priorities established by caregivers of CP children by level of severity and age, and to assess changes on performance and satisfaction on functional priorities reported by caregivers, in 6-month interval.

**Method:**

75 CP children, weekly assisted at Associação Mineira de Reabilitação, on physical and occupational therapy services. The following information was collected: gross motor function (Gross Motor Function Classification System-GMFCS) and functional priorities established by caregivers (Canadian Occupational Performance Measure-COPM). Data were collected in two moments, with a 6-month interval.

**Results:**

The main functional demands presented by caregivers were related to self-care activities (48.2%). Parents of children with severe motor impairment (GMFCS V) pointed higher number of demands related to play (p=0.0036), compared to the other severity levels. Parents of younger children reported higher number of demands in mobility (p=0.025) and play (p=0.007), compared to other age groups. After 6 months, there were significant increase on COPM performance (p=0.0001) and satisfaction scores (p=0.0001).

**Conclusions:**

Parents of CP children identified functional priorities in similar performance domains, by level of severity and age. Orienting the pediatric rehabilitation process to promote changes in functional priorities indentified by caregivers can contribute to the reinforcement of the parent-therapist collaboration.

## Introduction

Cerebral palsy (CP) is a health condition that primarily affects musculoskeletal functions and structures, resulting from damage to the brain in the prenatal, perinatal, or early childhood period^1^. These changes may have repercussions on the different ways of performing daily living activities, ranging from the need for total caregiver assistance to performing functional activities independently, even if in an alternative fashion and/or using assistive technologies^1^^,^ 2. In this context, current knowledge of neuromusculoskeletal manifestations and symptoms of this health condition is not sufficient to predict the functionality of the child in self-care activities, functional mobility, play, and school^3^^,^ 4. 

The literature has shown that the functional performance of a child with CP is not a direct consequence of the characteristics of the health condition. Mancini et al.^3^ analyzed the impact of motor function severity on the functional performance of children with CP. When comparing children with different motor function severity, children with moderate motor severity (level III of the Gross Motor Function Classification System [GMFCS]) demonstrated a functional repertoire similar to that of children with mild motor function severity (GMFCS levels I and II). In terms of independence, the moderate group (GMFCS level III) had similar results to the group of severely impaired children (GMFCS levels IV and V)^3^. These results are corroborated by Chagas et al.^4^, who analyzed the functional profile of children with CP according to classification systems of gross motor function severity (GMFCS)^5^^,^ 6 and manual function (MACS-Manuals Skills Classification System)^7^. They found that children classified as moderate in gross motor function (GMFCS level III) had a functional profile similar to that of children classified as mild (GMFCS levels I and II)^4^. However, considering the severity of the manual function, children with moderate impairment (MACS level III) showed greater functional similarity to children classified as severe (MACS levels IV and V)^4^. 

In addition to variability in the functional profile of children with CP across different levels of motor severity, nonlinearity can be observed regarding limitations in performing activities in different functional areas, such as self-care, mobility, and social function. Mancini et al.^3^ found that children with moderate severity (GMFCS level III) had a similar functional skills profile to that of children with mild severity (GMFCS levels I and II) in terms of self-care activities and social function, whereas in the area of mobility, these same children resembled children with severe motor impairment (GMFCS levels IV and V). These results illustrate the heterogeneity of functional manifestations of children with CP and underline the fact that a combination of factors, including physical, attitudinal, social, and technological characteristics, can influence the functional profile of a child in his/her daily routine^8^, restricting the exclusive predictive power of neuromusculoskeletal disorders. Thus, considering the variability of the functional limitations that can arise from CP, rehabilitation actions with measures of individual outcomes can promote the participation of children in their different life contexts. 

Collaborative actions between family and therapists are essential for developing individualized rehabilitation strategies that effectively promote the child's functionality^1^^,^ 9 ^-^ 13. Egilson^10^ analyzed parents' perceptions regarding rehabilitation actions for children with physical disabilities, and emphasized the desire reported by parents to be kept informed and to participate in the therapeutic decision-making process. Thus, parents seek information that is useful for improving the child's functional performance in daily living activities and they are concerned with the transfer of the learning acquired in the therapeutic environment to domestic and school contexts^10^. Hurlburt et al.^11^, investigating the characteristics of a child rehabilitation service, observed that incongruence between the perceptions of therapists and family members regarding the rehabilitation process could hinder the family's understanding regarding intervention, potentially reducing therapeutic results in terms of the child's functional performance. Øien et al.^13 ^explored parents' and professionals' perspectives about establishing meaningful goals for families of children with CP. The authors noted that the involvement of parents in establishing therapeutic goals could increase feelings of competence and participation and could contribute to the partnership between parents and professionals^13^. 

A central goal of rehabilitation is related to the promotion of the child's participation in significant life contexts. Therefore, it is important that therapists be aware of the priorities and needs of the child from the perspective of the caregiver, as, in terms of daily living, the parents are highly knowledgeable regarding the child's skills and needs^1^^,^ 9 ^,^ 14. Knowledge of these priorities by means of using instrumentation that captures functional demands can, therefore, help the therapist to develop individualized rehabilitation strategies that are meaningful and appropriate to the family's priorities. The main goal of this study was to identify functional goals established by caregivers of children with CP assisted at a rehabilitation center and to relate this information to the child's severity of CP and age. In addition, the study also sought to evaluate changes in performance and satisfaction reported by caregivers regarding the functional priorities over a period of six months. 

## Method

This retrospective longitudinal study reviewed the medical records of 75 children with CP who attended the Associação Mineira de Reabilitação (AMR) on a weekly basis in Belo Horizonte, MG, Brazil, during the period from July to December 2011. This study, along with the terms of free and informed consent, was approved by the Núcleo de Ensino e Pesquisa da AMR and the *Universidade Federal de Minas Gerais *(UFMG), Belo Horizonte, MG, Brazil, Research Ethics Committee (ETIC-02740203000-10). 

### Participants 

Participants were children with CP between 3 and 16 years of age, with a clinical diagnosis established by neurological examination, who attended weekly physical therapy and occupational therapy sessions. Children who were on sick leave during the collection period or who followed a different attendance regime, such as biweekly or monthly monitoring, were excluded. Children who missed three or more sessions during the reporting period were also excluded. 

### Instrumentation 

Initially, information was collected from medical records regarding children's GMFCS gross motor function severity level^4^^,^ 5. The GMFCS classifies gross motor function on five levels based on the sitting, standing and walking skills of the children with CP as well as their use of support devices and adaptive equipments^4^^,^ 5. At level I, the child is able to walk without difficulty in different environments and has skills such as running and jumping. At level II, the child can walk on stable surfaces but can have difficulties and may need support or equipment for long distances. Children at Level III make use of a support apparatus in indoor environments and a wheelchair in outdoor environments. Children at level IV have limited mobility and can propel a wheelchair. Finally, children at level V have severe motor impairment and require the constant use of a wheelchair and assistance^4^^,^ 5. 

Information regarding the functional demands established by the family were obtained using the Canadian Occupational Performance Measure (COPM)^15^. For this study, data were collected from children in two stages, with an interval of six months between data collection periods, via an interview with the child's caregiver. The interviews were held by the same examiners, occupational therapists previously trained to apply the instrument. 

The COPM is a standardized tool that assists therapists in intervention based on the priorities established by the client^15^. In the AMR's evaluation routine, the COPM is administered through interviews with caregivers. In this evaluation, there are scores of activity importance on a 10-point scale (1 = not important, 10 = very important) in different occupation areas (self-care, productivity, leisure)^15^. Caregivers were asked to list the five activities they felt were the most important and to rate the child's performance and their level of satisfaction with the way he/she performed each activity on a scale of 1to 10^15^. Studies report that the COPM has good validity and reliability^15^^,^ 16. Revaluation was performed with the same caregiver who responded to the first interview. 

### Intervention procedures 

Rehabilitation sessions were conducted at AMR and included weekly physical therapy and occupational therapy interventions. Frequency of rehabilitation sessions and children's treatment goals in each specialty were determined semi-annually in a discussion among the professionals serving the child. Intervention planning was based on data obtained from the application of the COPM. At the time of discussion, the professionals, knowing the parents' priorities as described by the COPM, established the intervention goals for the next six months of rehabilitation. Appointments were individual and lasted 45 minutes. Table 1 shows the appointment frequency in the physical therapy and occupational therapy services. 

**Table 1:** Frequency (%) of children assisted weekly at physical therapy and occupational therapy services at the AMR (Associação Mineira de Reabilitação), from July to December, 2011.

Number of weekly sessions*	Physical Therapy	Occupational Therapy
2	37 (49.3%)	15 (20%)
1	38 (50.7%)	60 (80%)

* Duration of each session: 45 minutes.

### Data analysis 

Frequency, percentage, and mean are used to describe the characteristics of children with CP, for age, gender, diagnosis, and GMFCS level data^4^^,^ 5. The five main functional activities mentioned by parents in implementing the COPM were categorized into groups of activities: personal care, mobility, play, school, socialization/communication, participation in household chores, and independence outdoors. In addition, chi-square tests were used to ascertain the association between the functional goals established by the caregiver in the COPM and the severity of motor impairment as well as between functional goals and age group. Chi-square tests were also used to investigate the association between the frequency of weekly sessions and clinically significant changes that, in the COPM test, correspond to two points or more between longitudinal measures^15^. The analysis of the changes in COPM scores over the six-month period was preceded by normality tests. The nonparametric Wilcoxon test was used because the data did not show a Gaussian distribution. The significance level adopted in all analyses was α=0.05. 

## Results

Table 2 presents the principal descriptive characteristics of gender, age, clinical diagnosis, and gross motor function level of the children in the study. 

**Table 2:** Description of children with cerebral palsy regarding gender, age, diagnosis, and gross motor function level, according to the Gross Motor Function Classification System (GMFCS).

Descriptive categories	Frequency (%)
Gender	
Male	45 (60%)
Female	30 (40%)
Medical diagnosis	
Spastic Quadriparesis CP	36 (48%)
Spastic Diparesis CP	14 (18.7%)
Dyskinetic CP	11 (14.7%)
Spastic Hemiparesis CP	8 (10.7%)
Mixed CP	4 (5.35%)
Ataxic CP	2 (2.6%)
Gross Motor Function (GMFCS) Level	
I	6 (8%)
II	15 (20%)
III	6 (8%)
IV	39 (52%)
V	9 (12%)
Age (years)	Values
Average age (standard deviation)	7.35 (3.28)
Maximum age	16
Minimum age	3
Age groups	
3-6 years old	31 (41.33%)
7-10 years old	29 (38.67%)
11-16 years old	15 (20%)

### Functional priorities and gross motor function severity 

Of the 278 demands reported, 134 (48.2%) referred to personal care activities, followed by school activities (19.78%), play (14.39%), and mobility (12.95%). Other demands included socialization activities, household chores, and independence outdoors (4.68%). 

Table 3 presents the functional priorities identified by the caregivers of children with CP during application of the COPM at different levels of gross motor function, by occupation area. It was observed that priorities relating to personal care were the most frequent at all levels of motor severity, except for children at GMFCS level V. Analysis of the relationship between priorities identified by the children's motor function level and occupational area revealed that parents of children classified as GMFCS V reported priorities relating to play as the most important (χ^2^=10.30, p=0.036). No significant associations between gross motor function severity and other occupation areas were observed. 

**Table 3:** Frequency (%) of activities (n=278) grouped in occupational areas listed as priorities by the caregivers of children with cerebral palsy from different levels of gross motor function according to the Gross Motor Function Classification System (GMFCS).

Occupational Areas		GMFCS Level				
		GMFCS I	GMFCS II	GMFCS III	GMFCS IV	GMFCS V
Personal Care	Feeding	4 (18.18%)	5 (8.19%)	2 (9.52%)	19 (12.94%)	3 (11.11%)
	Dressing	4 (18.18%)	17 (27.87%)	3 (14.29%)	26 (17.69%)	-
	Hygiene	2 (9.09%)	4 (6.56%)	2 (9.52%)	13 (8.85%)	-
	Taking a shower	3 (13.63%)	3 (4.92%)	1 (4.76%)	9 (6.12%)	3 (11.11%)
	Sphincter control	-	2 (3.28%)	3 (14.29%)	5 (3.40%)	1 (3.70%)
	Total of demands	13 (59.08%)	31 (50.82%)	11 (52.38%)	72 (49%)	7 (25.92%)
Mobility	Transfers		2 (3.28%)		7 (4.76%)	7 (25.93%)
	Locomotion	3 (13.63%)	3 (4.92%)	2 (9.52%)	11 (7.48%)	1 (3.70%)
	Total of demands	3 (13.63%)	5 (8.2%)	2 (9.52%)	18 (12.24%)	8 (29.63%)
School	Educational concepts	-	3 (4.92%)	1 (4.76%)	5 (3.40%)	-
	Pencil use	2 (9.09%)	7 (11.47%)	4 (19.06%)	18 (12.24%)	1 (3.70%)
	Use of other school Materials	1 (4.55%)	5 (8.19%)	1 (4.76%)	3 (2.04%)	-
	Attention	-	1 (1.64%)	-	3 (2.04%)	-
	Total of demands	3 (13.64%)	16 (26.22%)	6 (28.58%)	29 (19.72%)	1 (3.70%)
Play	Play Structure	-	-	-	6 (4.08%)	2 (7.41%)
	Play Interaction	-	-	-	3 (2.04%)	1 (3.70%)
	Hand use while playing	-	3 (4.92%)	-	7 (4.76%)	5 (18.53%)
	Attention	-	-	-	1 (0.68%)	1 (3.70%)
	Specific play	1 (4.55%)	2 (3.28%)	2 (9.52%)	4 (2.72%)	-
	Play positioning	-	1 (1.64%)	-	1 (0.68%)	-
	Total of demands	1 (4.55%)	6 (9.84%)	2 (9.52%)	22 (14.96%)	9 (33.34%)
Socialization	Socialization /Communication	1 (4.55%)	-	-	5 (3.40%)	2 (7.41%)
Household chores	Household chores	1 (4.55%)	1 (1.64%)	-	1 (0.68%)	-
Independence outdoors	Independence outdoors	-	2 (3.28%)	-	-	-

% were calculated regarding the total of functional demands per gross motor function level (GMFCS).

### Functional priorities and age groups 

Table 4 presents functional priorities identified by the caregivers of children with CP in different age groups. The activities with the greatest frequency of parental demand across all age groups were in the category of personal care. Analysis of the relationship between priorities identified according to age and occupational area revealed that children in the youngest age group (3-6 years) more often had priorities relating to mobility (χ^2^=7.35, p=0.025) and play (χ^2^=9.99, p=0.007) when compared to other age groups. No significant associations between age group and other occupational areas were observed. 

**Table 4:** Frequency of priorities (n=278) in different occupational areas with respect to the age group of children with cerebral palsy.

Occupational areas	Age group		
	3-6 years old	7-10 years old	11-16 years old
Personal care	46 (42.99%)	55 (50.92%)	33 (52.38%)
Mobility/transfers*	21 (19.63%)	6 (5.56%)	9 (14.27%)
Play**	22 (20.56%)	15 (13.89%)	3 (4.77%)
School	16 (14.95%)	25 (23.16%)	14 (22.22%)
Socialization/communication	2 (1.87%)	5 (4.63%)	1 (1.59%)
Household chores	0	1 (0.92%)	2 (3.18%)
Independence outdoors	0	1 (0.92%)	1 (1.59%)
Total of demands	107	108	63

* Significant association between age group and mobility/transfers (χ2=7.35; p=0.025);

** Significant association between age group and play (χ2=9.99; p=0.007).

### Functional outcome in children after six months 

**Figure 1:**
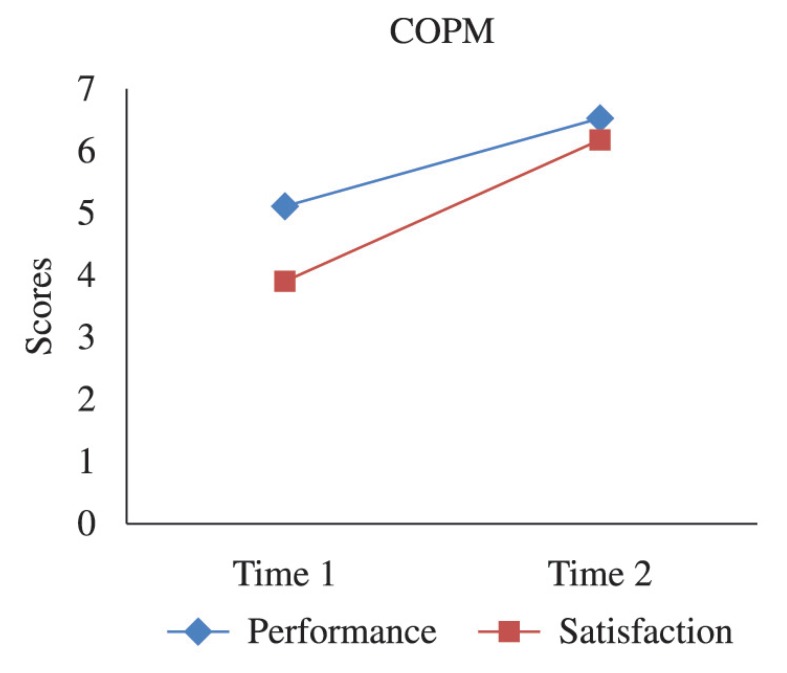
Changes in the performance (p=0.001) and satisfaction scores (p=0.0001) of parents regarding functional priorities (COPM test) on a 6-month interval (time 2 - time 1).

In the six-month period between the two stages of data collection, there was a significant increase in COPM score, in terms of both performance (p=0.0001) and satisfaction (p=0.0001) (Figure 1). No association was found between the frequency of sessions (twice or once per week) and the magnitude of clinically significant gains in COPM performance scales (p_Physical therapy_ = 0.197, p_Occupational_ _Therapy_ or satisfaction scales (p_Physical therapy_ = 0.514, P _Occupational Therapy_= 0.221).

## Discussion

This study presents the main functional priorities indicated by caregivers of children with CP in different occupation areas and functional changes over a period of six months. Personal care activities were more important for parents and caregivers, followed by school activities and play. Priorities related to play occurred mainly in children with greater motor severity (GMFCS level V) and in the youngest age group (3-6 years). There was also an increase in performance and satisfaction scores in functional activities deemed important by caregivers over the period of six months. 

With regard to functional demands of the COPM, the priorities identified by parents were principally personal care activities, such as dressing, feeding, bathing, hygiene, and toilet training. These results are corroborated by data from Chiarello et al.'s study^2^, which also found that personal care activities were important for all parents of children in their study^2^. The focus on personal care activities reflects parents' expectations for the child to obtain greater independence, autonomy, and efficiency in carrying out those activities^2^. According to Barrett and Kielhofner^17^, performing personal care activities ensures the satisfaction of basic needs and independence in the home environment^17^. Moreover, the child's performance in these activities allows him/ her to experience independence and to develop skills for participating in other performance areas, such as education, work, and leisure^2^. 

Functional mobility activities were not the main demand of the parents of children in this study. Although mobility is related to changes in neuromusculoskeletal function and structures in children with CP, it was not reported by caregivers as a priority outcome. These results are contrary to the results presented by Knox^18^, who conducted a retrospective study by reviewing the records of 121 children with CP to determine the functional performance interests of parents as reported to the professionals. In Knox's study, the main demands identified by parents of children with CP at GMFCS level I focused on standing, walking, and manual function, while at levels II to IV, the main demands were related to activities such as standing and walking^18^. Parents of children classified as GMFCS level V prioritized mobility (i.e. sitting, mobility on the ground) and communication skills^18^. The differences between the results of this study and the results reported by Knox^18^ could be attributed to different characteristics of the instrumentation used and age associated with the participants' motor function severity. The present study used the COPM to discuss the functional demands of the child's daily routine considered important by parents, whereas Knox's^18^ used data from medical records to identify parents'priorities. In addition, the majority of Knox's study participants^18^ were children at GMFCS IV and V motor severity levels (57%) and were younger than 6 years (68%), whereas in th present study, most children had GMFCS severity level IV and were aged over seven years (58.67%). In the present study, mobility demands in the 3-6 year age group were more frequent than mobility demands in older age groups. According to Rosenbaum et al.^19^, children with greater motor severity tend to stabilize their acquisition of gross motor function after 5 years of age. It is possible therefore, that the parents of the children in this study, especially of children over the age of 7, were aware of the child's gross motor function stability and therefore prioritized meeting personal care, play, and school demands, focusing on the greater participation of their children in these functional domains. 

Demands related to play were identified as most important by caregivers of young children and children with severe motor impairment. Considering the importance of play as a primary childhood activity^20^, this outcome illustrates an important functional goal for therapeutic intervention^21^. During play, the child has the opportunity to discover relationships among objects, people, and actions, to explore the environment, and to develop social and occupational roles^20^. Pfeifer et al.^22^, in evaluating the spontaneous play skills of children with CP between 3 and 6 years of age, described characteristics of spontaneous play, such as starting a game on their own, exploring a toy, preparing and sequencing the activity, and pretending play. In this study, children with greater motor severity had significant difficulties in developing more sophisticated playful actions and had a limited repertoire of symbolic play^22^. Mobility restrictions might, therefore, hinder participation in certain play activities, for example, those that require motor skills or more complex cognitive functions, without completely restricting the recreational engagement of these children. It is important to identify alternative forms and adaptations that might promote the engagement of children with CP at different motor function severity levels in recreational experiences and interactions with parents and other children^23^. 

In the present study, there was improvement in the children's performance and the parents' satisfaction with regard to functional objectives over a six-month period. This result illustrates the importance of using standardized instrumentation to document functional gains defined as priorities for families of children with CP and also to serve as a facilitator in the family-therapist collaborative relationship. Parental involvement in decision-making about therapeutic goals to be achieved is considered an important element in the rehabilitation process^1^^,^ 9 ^-^ 13. Øien et al.^13^studied parents' and professionals' perceptions about setting goals for the rehabilitation of children with CP and stressed that the family's establishment of priorities contributed to the ability of parents to position themselves and communicate the needs and preferences of the children and family. This cooperative action with parents in the development of therapeutic strategy enhances the practice of these goals in the family context^13^. Anderson and Hinojosa^24^ claimed that intervention with the child could be optimized through the development of a positive relationship between therapists and family members. In this partnership, professionals must recognize the role of parents in the therapeutic process, understand the characteristics of the parent-child relationship, and adapt actions by guiding therapy in an effective collaboration with parents for the benefit of the child's rehabilitation process^24^. By interviewing caregivers to identify important functional goals for the family, professionals can make therapy more effective and meaningful for the child and family^25^. 

### Limitations of the study 

This study showed improvements in parents' perception of children with CP in terms of the functional objectives prioritized over a six-month period. However, as the study was retrospective, it was not possible to control of the training intensity of each functional demand identified as important by parents. Thus, it is not possible to say that the observed changes were solely the result of rehabilitation actions. Future prospective and controlled studies might elucidate the effects of interventions focused on specific needs identified by caregivers of children with CP. 

### Clinical implications 

The present study described and characterized the major functional priorities of caregivers of children with CP treated at a rehabilitation center. The most frequent priorities were related to personal care activities. There was improvement in functional performance and parental satisfaction with functional objectives considered important. This information underlines the importance of using an individualized functional measure focused on the expectations and priorities of the family to understand the child's daily routine and to establish meaningful interventions in his/her life context. 
